# Case study: creating an ‘AI for Academic Writing Skills’ induction session for postgraduate life science courses

**DOI:** 10.1042/ETLS20253026

**Published:** 2025-12-18

**Authors:** Jennifer Carter, Anne Ferrey, Hubert Lam, Kelly Webb-Davies, Damion Young, Barbara Zonta, Delia O' Rourke

**Affiliations:** 1 Nuffield Department of Population Health, University of Oxford, U.K.; 2 Health Data Research, University of Oxford (HDRUK-Oxford), U.K.; 3 Nuffield Department of Primary Care Health Sciences, University of Oxford, U.K.; 4 AI Competency Centre, University of Oxford, U.K.; 5 Medical Sciences Division, University of Oxford, U.K.; 6 Department of Pharmacology, University of Oxford, U.K.; 7 Nuffield Department of Medicine, University of Oxford, U.K.

**Keywords:** academic writing, ethical use, generative AI, induction session, postgraduate taught

## Abstract

Student use of generative artificial intelligence (GenAI) technology in university is as ubiquitous as it is controversial, and students and staff need guidance on how to use it effectively, responsibly, ethically and critically. In this case study, we present the conception, design and evaluation of a pilot ‘AI for Academic Writing Skills’ induction session designed for postgraduate taught (PGT) students in Medical Sciences. The induction session was designed by an institution-wide collaboration between Master of Science (MSc) programme directors, senior learning technologists and senior business technologists in AI competency to provide approximately 100 PGT students with structured, practical training on ethical GenAI use in academic writing. To ensure equitable access for all students in the pilot, this initiative integrated ChatGPT4 via a paid application programming interface into the institution’s virtual learning environment, Canvas, ensuring all the students had access to the latest version of the chatbot. Evaluation of the ‘AI for Academic Writing Skills’ induction session demonstrated that 100% of survey respondents rated the training positively, 86% found the academic writing lecture beneficial, and 100% found the lecture on general GenAI skills helpful. Furthermore, 82% appreciated interacting with the chatbot in group work, and 70% reported significantly reduced uncertainty about using GenAI in their academic work.

This case study details our approach, which first surveyed students to assess current levels of engagement and confidence with GenAI tools. Based on these findings, we developed a scalable, evidence-based induction session on the ethical use of GenAI in academic writing. This report describes the process of creating this training, its impact on student confidence, and our reflections on how we will continue to refine the programme in future academic years.

## Introduction

The number of postgraduate taught (PGT) programmes in the UK has grown significantly [1,2], attracting a diverse cohort of students with a wide range of prior knowledge, professional and academic experience, and cultural backgrounds. This diversity enriches the learning environment, yet PGT students also face a combination of unique challenges. These include the short, intensive timeframe of their courses, managing concurrent work and family responsibilities, financial pressures [3,4], and navigating the significant diversity in academic conventions [5].

The recent emergence of generative artificial intelligence (GenAI) tools has introduced a new layer of complexity [6]. Although UK universities have issued guidance on GenAI use [7], we observed that PGT student uncertainty persisted, creating a general sense of unease and sometimes anxiety. Despite this, the ethical and effective use of GenAI tools is now an essential skill for students. A core skill for all PGT students is academic writing, and proficiency in this area often underpins success in PGT programmes. However, the diversity of academic writing experience within incoming cohorts can lead to inequalities, particularly for those whose first language is not English [8,9], those with limited exposure to academic writing conventions, or neurodivergent students [10]. While many universities provide dedicated writing skills support [5,11,12], it is often primarily aimed at undergraduates or postgraduate research students. Research suggests that with insightful prompts, GenAI can provide valuable feedback and iteratively improve writing quality [13]. Research focused on postgraduate experiences confirms that students recognise GenAI’s importance in enhancing academic skills, such as improving writing, translation and facilitating self-directed learning [14]. Students use GenAI to assist with content comprehension and understanding research articles [15]. Furthermore, the potential for GenAI to act as a linguistic aid is particularly critical, as it can promote digital equity by providing robust language and writing support for international and non-native English-speaking students [15]. However, there is a lack of evidence regarding how PGT students and teaching staff can engage ethically with these tools in academic writing [16].

To address these gaps, members of the Oxford Postgraduate Teaching Network, a community of practice for PGT staff [17], initiated a project to support students in developing academic writing skills with the ethical use of GenAI tools. The use of GenAI in academic writing has been controversial, sparking debate among both students and academics regarding its role in knowledge acquisition, assessment, authorship and data analysis [18]. The key concerns that underpin the need for structured guidance for students include integrity, ethics and competency.

1. **Integrity:** AI tools have been shown to have issues with plagiarism, hallucinations (fabricated or false information) and producing inaccurate or fabricated references [19] .
2. **Ethics:** There is little clear guidance for scholars outlining the legitimate and ethical ways that GenAI could be used to support academic literature production. Students often experience uncertainty and negative emotions, such as stress and guilt, due to unclear guidelines on proper usage and attribution [14].
3. **Competency:** Educational experts argue that institutions must move beyond prohibiting GenAI and instead create structured opportunities for students to learn to use the tools critically, ethically and effectively, focusing on fostering core digital competencies [15]. GenAI should be used to augment, not replace, an author’s intellectual contribution [20] .

We describe how we established a responsive PGT-focused induction session in academic writing to address these concerns and uncertainties. Our induction session aimed to highlight benefits and risks of using GenAI tools to assist in academic writing, provide clear guidance on ethical use, and set and align students’ and staff expectations on GenAI usage, particularly in the context of assessments.

## Objectives

The objectives of our project, which began in early 2024, were to:

1. Assess current postgraduate student engagement with, and self-efficacy in, the use of GenAI tools for academic writing.
2. Develop an induction skills training session to help students improve their academic writing skills.
3. Establish and communicate a unified message on the ethical use of GenAI for academic work to both students and staff in Medical Sciences PGT programmes.

**Figure 1:**
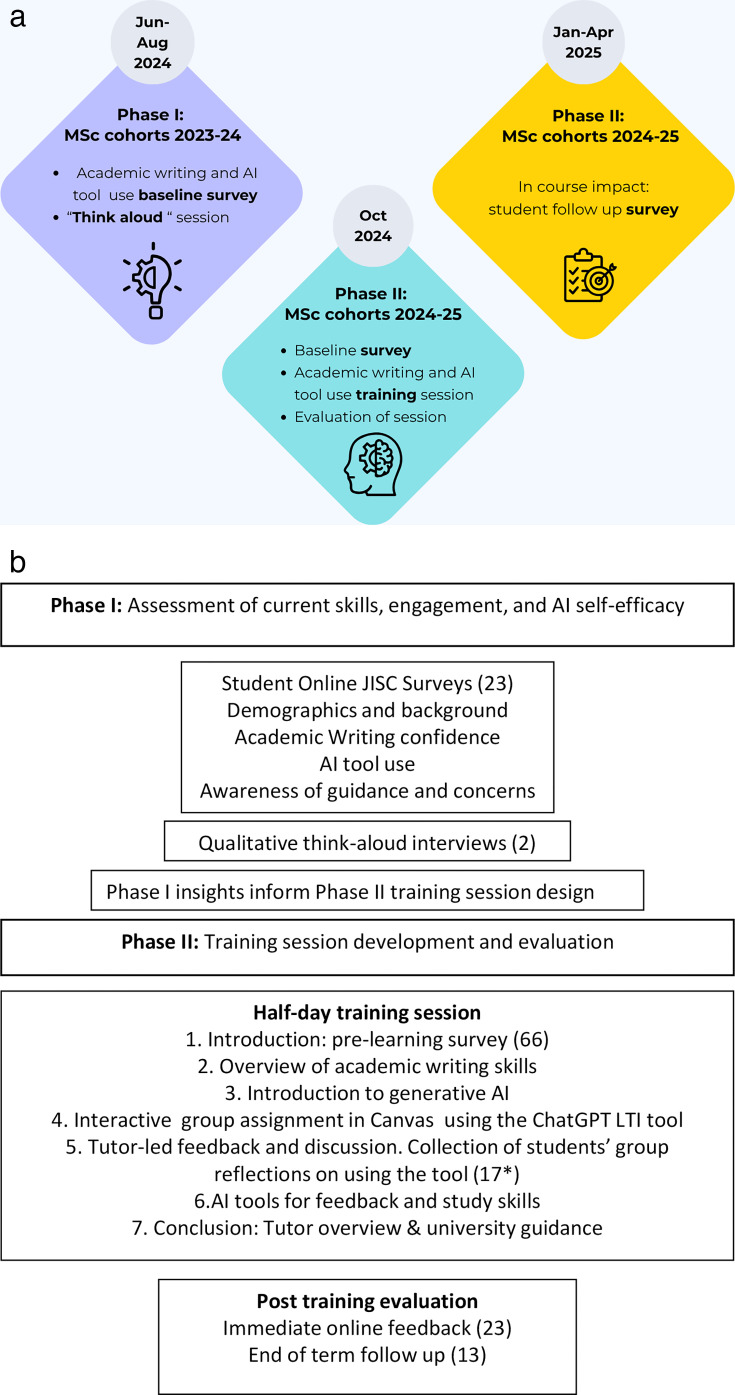
Study design for ‘Assessing and developing student use of GenAI for academic writing’. (a) Overview of the project phases. The MSc cohorts for 2023–24 and 2024–25 were from four 1‑year MSc programmes in the Medical Sciences Division. (b) Further detail of the methods and activities in each phase. Numbers in brackets represent the number of students who gave quantitative and qualitative feedback at each stage of the project.

This case study employed a two-phase design to develop and evaluate the GenAI skills training. The findings from Phase I directly informed the content and training session of Phase II. The methods are summarised in the flowchart in Figure 1b.

## Methods

### Phase I: assessment of current academic writing skills, engagement and self-efficacy with GenAI tools

The first phase focused on understanding students' existing confidence in academic writing and engagement with GenAI tools. We conducted a pilot study across four 1-year, in-person MSc programmes in Oxford’s Medical Sciences Division, in the final term of their taught programmes from June to August 2024, MSc cohorts 2023–24 (Figure 1). Over this period, students were conducting research projects and writing their dissertations.

### Student surveys

We distributed a JISC-based online survey [21] to all PGT students on the four MSc programmes (MSc cohorts 2023–24). The survey gathered data on prior academic backgrounds and perceived confidence in academic writing at the start and end of their academic programmes throughout 2023–24, and enquired as to which resources or activities had been most useful in developing their academic writing. It then focused on students' self-efficacy with GenAI tools, exploring usage patterns, the specific tools they employed, and their confidence in using GenAI tools. The final section examined their awareness of university guidance and any concerns they had regarding usage of GenAI tools to support their academic writing. Key questions and response data are collated in the Supplemental file. Free text quotes were not independently analysed for this case study, and illustrative quotes were chosen by the project team and gathered in five themes.

### Qualitative student interviews

Two PGT students who completed the online JISC survey described above volunteered for an optional qualitative ‘think-aloud’ interview. Participants were given a generic 300-word essay question and asked to use a GenAI tool to structure and/or write the essay during the ‘think-aloud’ session [22]. Participants were instructed to vocalise their thoughts and actions as they used the tool to structure the essay and then to reflect on the experience of using a GenAI tool to answer an essay prompt. Audio recordings of the entire session were made using Microsoft Teams. The ‘think-aloud’ element of the sessions lasted up to 20 minutes, with the entire interview lasting approximately 40 minutes. Participants received a £10 book voucher for their time and travel. Audio recordings were transcribed by Microsoft Teams and checked by the researcher. Transcriptions were then explored using a modified ‘One Sheet of Paper’ Method [23] to summarise key themes regarding student engagement with GenAI. Due to low participant numbers, no further thematic analysis was conducted. Nonetheless, the ‘think-aloud’ output informed the design and development of activities for the ‘AI for Academic Writing Skills’ training session. Overall, quotes from the MSc 2023–24 cohorts’ survey and the ‘think-aloud’ session were used to support our identification of five general themes (see Phase I Results)

### Phase II: training development and evaluation

Considering the results of Phase I, the second phase involved designing, implementing and evaluating an ‘AI for Academic Writing Skills’ induction training session for the incoming 2024–25 cohort of students. The training session took place during induction week in October 2024 and approximately 100 incoming students from the four pilot MSc programmes attended a half-day, in-person session. Before we began the session, 66 students completed an online survey similar to the one used in Phase I to provide baseline data on academic writing and self-efficacy in using GenAI tools.

### Integration of ChatGPT4 into the virtual learning environment, Canvas

To ensure that all students had equitable access to the most up-to-date version of an AI chatbot, ChatGPT4 was integrated using a Learning Tools Interoperability (LTI)-GPT link in the Canvas navigation menu. This tool acted as a wrapper for the publicly available ChatGPT. The students were given the following instructions on the Canvas page:

#### Use the LTI-GPT link on the left to help with your academic writing skills

Remember:

1. Sources can be hallucinated and should not be relied upon.
2. Review all references, or use a reputable literature search tool for research, reviews, primary sources and images to cite/adapt.
3. Define your audience in your prompts (i.e. messages you send).

This tool is a wrapper for the publicly available ChatGPT tool.

All learning materials, links to surveys, assignments and lecture recordings were shared with students via the Canvas course ‘AI for Academic Writing Skills’.

### AI for Academic Writing Skills: training session structure

The 4-hour training session during induction week in October was structured as follows:

- **Introduction:** Students were encouraged to complete an anonymous pre-learning survey exploring their confidence in academic writing and their use and confidence with GenAI tools. The goal of the session was to explore ‘How do we use generative AI tools to help support learning and academic writing in appropriate and effective ways?’
- **Overview of academic writing skills:** A 45-minute lecture, covering
  - Academic language and style.
  - Planning and structure.
  - Referencing and plagiarism.
  - Critical writing.
- **A short introduction to generative AI:** A 30-minute lecture, covering
  - GenAI – what is it? and how does it work?
  - GenAI – limitations and strengths.
  - GenAI personas as analogies for how to view and interact with GenAI. The stranger, the intern, the translator, the tutor.
  - Why are you here? Using evaluative judgement.
- **Interactive Assignment:** Students worked in small groups on an interactive assignment using a university-approved chatbot in Canvas to write an essay outline and short introduction (less than 300 words). Students then completed a short reflection survey on their experience of this task, exploring prompt effectiveness and potential for bias.
- **Feedback & Discussion:** After reviewing students' plans and essays, tutors led a shared discussion informed by the submitted work, student reflections and feedback collected through a brief survey.
- **Advanced GenAI Use:** Two additional 20-minute sessions demonstrated how to use AI to provide writing feedback and enhance study skills.
- **Conclusion:** The session concluded with an overview of GenAI’s helpfulness and limitations, followed by the official university guidance on GenAI.

### Post-session evaluation

Immediately following the training session, students completed a short online evaluation form to provide feedback. A follow-up survey was distributed in January 2025 to measure long-term impact of the induction session.

## Results

### Phase I: assessment of current academic writing skills, engagement and self-efficacy with GenAI tools for 2023–24 cohort

**Figure 2:**
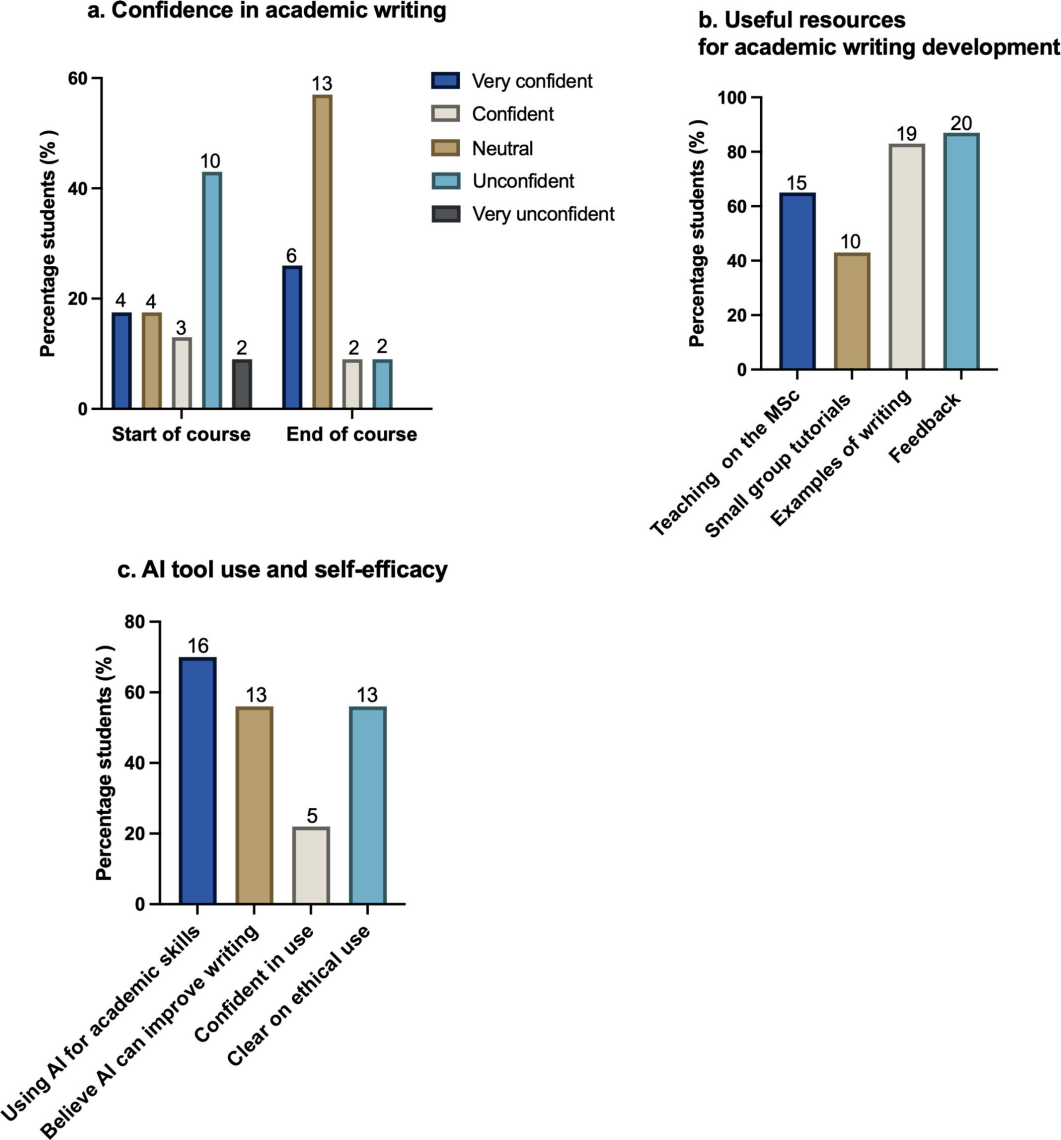
MSc cohorts 2023–24 Phase I study-survey, August 2024. We surveyed students from four MSc programmes at the end of the academic year, as they were writing up their dissertations. Twenty-three students responded to this survey. *n*=23. **(a)** Confidence in academic writing. We asked students to reflect back to the start of the programme and give an estimate of their confidence in academic writing (start of the course). “At the start of your current MSc course, what was your confidence level in essay and extended academic writing (e.g. literature reviews and/or dissertations)?” We then asked, “What is your current confidence level in essay and extended academic writing?” (End of course). **(b)** Useful resources for academic writing development. We asked students, “What resources have you found most useful in developing your academic writing since the start of your MSc course? (Select as many as applicable)”. **(c)** AI tool use and self-efficacy. We asked students about their use of AI tools in academic writing. “Are you using or have used AI-powered tools for academic writing? To what extent do you believe using AI-powered tools can help you improve your overall academic writing skills? How would you rate your general confidence in using AI-powered tools to improve your academic writing?”

In the summer term of 2024, as the 2023–24 cohort of MSc students were preparing their dissertations, we asked them to reflect back to the start of their MSc programme and give an estimate of their confidence in academic writing (Figure 2a ‘Start of course’) *– “*At the start of your current MSc course, what was your confidence level in essay and extended academic writing (e.g. literature reviews and/or dissertations)?” We then asked, “What is your current confidence level in essay and extended academic writing?” (Figure 2a ‘End of course’) – students’ perceived confidence in academic writing skills had improved from the start of the academic year. The percentage of students who were unconfident or very unconfident dropped from 52% at the start of the academic year to just 9% (Figure 2a). Conversely, those who were very confident or confident increased from 35% to 82% (Figure 2a).

We next asked students, “What resources have you found most useful in developing your academic writing since the start of your MSc course? (Select as many as applicable)” The most useful resources for developing academic writing included feedback on academic writing (87%), examples of academic writing (83%), teaching on their MSc (65%), and small group tutorials (43%) (Figure 2b). This feedback demonstrated that academic skills training across the MSc programmes was working effectively to develop students' writing skills.

However, for many of the 2023–24 cohort of students, their self-efficacy with GenAI tools was low. We asked students the following questions: “Are you using or have used AI-powered tools for academic writing? To what extent do you believe using AI-powered tools can help you improve your overall academic writing skills? How would you rate your general confidence in using AI-powered tools to improve your academic writing?*”* While 70% of students were using GenAI for academic tasks and 56% believed these tools could improve their writing, only 22% felt confident in their use (Figure 2c). Just over half of students (56%) were clear on when and where they could ethically use GenAI (supplementary file key survey questions and responses).

The qualitative think-aloud interview and feedback from the survey identified five general themes that we have used to collate quotes from students. These were

1. Plagiarism
2. Deskilling research and writing skills
3. Credibility and accuracy of information from Gen AI
4. Unsure how to construct effective prompts
5. Request for clear guidance, examples, resources and training.

Feedback from Phase I, 2023–24 cohort suggested that students were reluctant to use GenAI tools due to lack of knowledge about allowable use and worries about plagiarism. Participants did not want to “rely on it as a crutch*”*, preferring “to be in control of [their] own work” and developing their own skills. They also flagged the quality of outputs from the GenAI – “I would double check that [it is] correct”.

Student concerns focused on issues of plagiarism, inaccuracies (hallucinations), losing their personal writing style and becoming overly dependent on the technology (for themes and responses, please see Supplementary Table S1). This feedback directly informed the design of our Phase II induction training session, which students requested should provide hands-on practice, guidance on effective prompting and explicit examples of appropriate and inappropriate use to build confidence and reduce anxiety about academic misconduct.

### Phase II: training and evaluation

Using the findings from the outgoing 2023–24 MSc cohorts, we developed and delivered a 4-hour ‘AI for Academic Writing Skills’ induction training session for incoming students in the 2024–25 cohorts (Figure 2). A pre-workshop online survey of incoming students from the same four MSc programmes echoed the Phase I findings.

**Figure 3:**
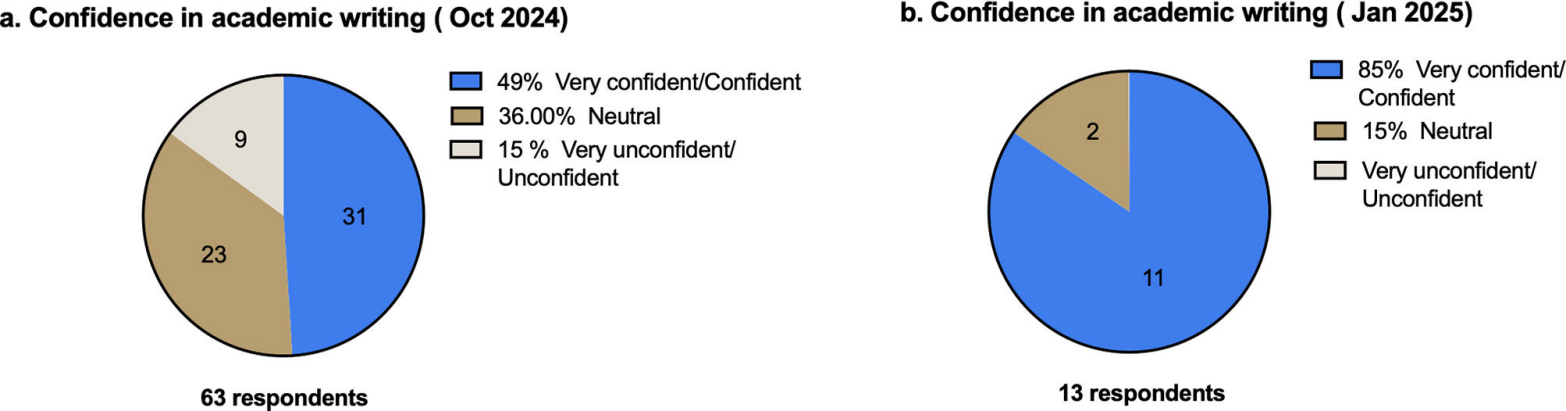
MSc cohorts 2024-25: Phase II study-survey data on confidence in academic writing. (a) Before completing the ‘AI for Academic Writing Skills’, we asked students “What is your current confidence in academic writing?” *n*=63. (b) Three months after the training, we asked students “What is your current confidence in academic writing?” *n*=13.

In Phase II, for incoming 2024–25 students before the training session, 49% of students were confident or very confident in academic writing, whilst 15% were not/not at all confident (Figure 3a); and 29% were very confident/confident of using GenAI-powered tools to improve academic writing, whilst 36% were not/not at all confident, with 35% neutral (supplementary data, key survey questions and responses).

During the workshop, a small-group activity required students to use the Canvas embedded LTI chatbot to write an essay outline and short introduction. We provided a series of reflection questions to students, including questions on accuracy, bias, outdated content and how different prompts changed the output. These were designed to help students engage critically with the output from GenAI and to help them develop prompt skills. For questions, see supplementary file, key survey questions and responses.

We collected reflections from 17 groups, which highlighted both the advantages and pitfalls of GenAI assistance. Students found GenAI tools valuable for initiating academic writing, particularly for generating outlines, structuring introductions and brainstorming. They viewed GenAI as a tool for efficiency and for overcoming initial writing blocks. However, the data also revealed significant pitfalls. Students noted the GenAI’s tendency towards vagueness, its unreliable referencing, its potential for inaccuracies and its difficulty with concise academic language.

### Evaluation and outcome of the induction session

Evaluation feedback from the induction day event was overwhelmingly positive (100% of respondents, *n* = 23, rated it positively). 100% found the session on general GenAI skills helpful, 86% found the academic writing portion beneficial, 82% appreciated the hands-on interaction with the integrated ChatGPT tool, and 70% reported a significant reduction in their uncertainty about using GenAI.

Qualitative feedback emphasised the programme’s practical and interactive approach. One student commented, “I liked the clarity about how we can actually utilise AI for academic writing. It is helpful to have it acknowledged as a tool rather than not discussed...” Others noted, “Really helpful introduction to AI! Also nice that is was similar MSc programs coming together. Appreciated the session towards the end on reviewing an essay using different prompts, and getting it to explain things to us in different ways”. “It was nice to try out the tools in the lecture and get feedback”. By January 2025, following the induction session, 85% of students were confident/very confident in academic writing (Figure 3), 70% were confident/very confident in using GenAI-powered tools to improve academic writing, 15% were not/not at all confident, and 15% were neutral (supplementary file, key survey questions and responses).

Following the training, at the end of the first term, tutors on one MSc programme noted anecdotally that students were more open in acknowledging their use of GenAI, suggesting that the training had encouraged transparency. The same tutors also observed anecdotally that while some essays still lacked in-depth research and critical analysis, they noted that most showed improved clarity, structure and organisation.

However, continued usage of the Canvas embedded ChatGPT LTI was low. This aligned with anecdotal verbal student representative feedback from one of the MSc programmes, reporting that students were anxious about using the Canvas-embedded chatbot due to being unsure if their usage would be tracked in Canvas.

### Suggestions for improvement in future training

Students provided helpful suggestions for future sessions, including more focused guidance on prompt engineering, clearer instructions on citing GenAI, more specific essay writing examples, additional practice in addressing GenAI ‘hallucinations’ and verifying information, and information on a wider variety of GenAI tools (supplementary material, themes and responses, ).

## Discussion

The ‘AI for Academic Writing Skills’ induction proved to be an effective model for integrating ethical GenAI use into postgraduate training. By first assessing student confidence and then delivering a hands-on, interactive session, we directly addressed existing uncertainties and inequalities surrounding these tools. The innovative deployment of a Canvas-embedded ChatGPT LTI tool was designed to ensure secure and equitable access, but interestingly, most students chose not to use this integrated chatbot, perhaps reflecting anxiety about GenAI usage being tracked in the virtual learning environment.

This project serves as a proof of concept, demonstrating that practical, hands-on training during a short induction period can effectively build skills and contribute to increased confidence in a rapidly evolving digital learning landscape. The positive feedback and significant reduction in student uncertainty validate this approach, though we cannot formally attribute the increased confidence to the workshops alone, as many MSc students also attend additional skills training that may have contributed to their development. The initiative has not only provided clear guidance, but we hope it has also fostered transparency in GenAI usage among students.

As GenAI continues to change the higher education environment, it is essential to align and maintain student and staff expectations. Since students' perceptions and usage of GenAI are changing annually [24,25], our training will be updated each year and expanded to include guidance on its use in all assessments. Our future sessions will align with updated institutional guidance for both students and academics. We will also frame our guidance around suggested criteria for ethical use and acknowledgement in academic research and authorship [19,20]. These criteria are built on the foundations of academic integrity, honesty and transparency and include a guarantee of human vetting, human contribution, acknowledgement and a clear statement describing the use of GenAI tools.

This case study lays a foundation for the development of key induction training sessions for students on GenAI tools and academic writing and their ethical and effective integration into writing instruction. We will continue to explore how GenAI tools can support inclusive teaching and learning for all students, including those with English as an additional language and neurodivergent students [13,26]. Ultimately, helping us to design MSc programmes that remove barriers to learning and support PGT student development and well-being [27,28].

**Summary**

- **The importance of the field:** The rapid emergence of generative artificial intelligence (GenAI) tools presents both significant opportunities and challenges for academic writing and integrity [29]. Addressing student uncertainty and ensuring ethical, effective integration of GenAI into academic practice is necessary.
- **A summary of the current thinking:** While GenAI tools hold tremendous potential, their use by university students requires clear ethical guidelines, focusing on transparency, substantial human contribution and rigorous vetting of GenAI output to mitigate risks such as plagiarism, hallucination and inaccurate references. GenAI tools should augment, not replace, academic intellectual contribution.
- **Comments on future directions:** The ‘AI for Academic Writing Skills’ induction provides a foundation for future initiatives. Future directions include assessing the long-term impacts of GenAI tool usage, exploring the effectiveness of specific GenAI tools, optimising their integration into writing instruction, and investigating their role in developing critical thinking skills. Continued discussion within the academic community is essential to ensure that the ethical application of these tools remains consistent with the evolving state of the technology. There is also potential to broaden the audience for the induction training to include postgraduate research and undergraduate students.

## Supplementary material

online supplementary table 1.

## Open Access

This article has been published open access under our Subscribe to Open programme, made possible through the support of our subscribing institutions, learn more here: https://portlandpress.com/pages/open_access_options_and_prices#conditional

## Abbreviations

AI: artificial intelligence
GenAI: generative artificial intelligence
LTI: Learning Tools Interoperability
PGT: postgraduate taught
