# Supplementary material for: Case study: creating an ‘AI for Academic Writing Skills’ induction session for postgraduate life science courses
Source: Emerg Top Life Sci. 2025 Dec 18;9(4):ETLS20253026. doi: 10.1042/ETLS20253026 (PMC12794324; doi:10.1042/ETLS20253026)
Supplement: online supplementary table 1. [file ETLS-9-4-ETLS20253026-s001.docx]

**Supplementary Table 1**.

**Themes from Phase I ‘think aloud’ and student feedback from 2023-24 cohort in response to the questions indicated.**

| **Theme** | **Representative Quotes** |
| --- | --- |
| **1. Plagiarism Risks**  Question: What concerns, if any, do you have about using AI-powered tools in your academic work?  *(Note: 6/14 replies highlighted plagarims)* | *Plagiarism and falling afoul of academic integrity guidelines, as well as incorrect statements generated by the AI that hamper my own work.*  *Concerned about plagiarism and citation errors. Plagiarism/concerns of academic integrity.* |
| **2. Deskilling & Loss of Voice** *(Fears of losing research/writing skills)*  Question: What concerns, if any, do you have about using AI-powered tools in your academic work? | *I don’t want to rely on it as a crutch.*  *I would be worried about losing my own writing style or having my ideas changed. I would also be concerned about losing control/ownership of the writing I produce."*  *Developing a dependency on it (i.e., not developing my own writing skills).*  *to be in control of own work* |
| **3. Credibility & Accuracy** *(Trust in AI-generated information)* **Question**: What concerns, if any, do you have about using AI-powered tools in your academic  work? | *I really don't trust their content… [I] don't need anything that would create a false narrative that I would waste my time trying to confirm.*  *I would double check that [it is] correct* |
| **4. Uncertainty on Prompting** *(How to construct effective prompts)* **Question** : What further guidance, if any, from the University on the  use of AI tools would you welcome? | *How to [ask] AI right questions?*  *.* |
| **5. Requests for Guidance & Training**  *(Note: 8/23 replies in 2023-24 survey)*  Question : What further guidance, if any, from the University on the use of AI tools would you welcome? | *Short courses or workshops on how to use them, similar to the mandatory research ethics course.*  *Explicit guidance to give me confidence that I will not get in trouble for using AI writing tools in specific ways.*  *A course on the tools available. I did not realise the array of tools out there beyond GPT*  *Session(s) on approved use of AI tools.*  *Examples of when the use of AI is considered unethical or inappropriate. These concepts are quite subjective and may be interpreted differently by the students.*  *If you're writing a long piece, where do you acknowledge use of AI tools?*  *Clear acknowledgement is unclear in this instance; if a mathematician uses a calculator they do not cite Casio as an*  *author in their latest published proofs.* |
